# Association of Sirtuin Gene Polymorphisms with Susceptibility to Coronary Artery Disease in a North Chinese Population

**DOI:** 10.1155/2022/4294008

**Published:** 2022-02-18

**Authors:** Xingfa Song, Haidong Wang, Chao Wang, Guangquan Ji, Pei Jiang, Donglou Liang, Xiaojing Wang

**Affiliations:** ^1^Department of Pharmacy, The Affiliated Lianyungang Hospital of Xuzhou Medical University/The First People's Hospital of Lianyungang, Lianyungang, China; ^2^Department of Pharmacy, Hainan General Hospital/Hainan Affiliated Hospital of Hainan Medical University, Haikou, Hainan, China; ^3^Science and Technology Department, The Affiliated Lianyungang Hospital of Xuzhou Medical University/The First People's Hospital of Lianyungang, Lianyungang, China; ^4^Department of Pharmacy, Jining First People's Hospital, Jining Medical University, Jining, China

## Abstract

**Aims:**

Coronary artery disease (CAD) represents the leading cause of death worldwide. Accumulating evidence also suggests that sirtuins (SIRTS) have been associated with CAD. The present study was aimed at investigating the association between 12 gene polymorphisms for SIRTs and the development of CAD in a Chinese population.

**Materials and Methods:**

12 SNPs (rs12778366 (T > C), rs3758391 (T > C), rs3740051 (A > G), rs4746720 (C > T), rs7895833 (G > A), rs932658 (A > C) for SIRT1, rs2015 (G > T) for SIRT2, rs28365927 (G > A), rs11246020 (C > T) for SIRT3, rs350844 (G > A), rs350846 (G > C), and rs107251 (C > T) for SIRT6) were selected and assessed in a cohort of 509 CAD patients and 552 matched healthy controls for this study. Genomic DNA from whole blood was extracted, and the SNPs were assessed using MassARRAY method.

**Results:**

TT genotype for rs3758391 and GG genotype for rs7895833 of SIRT1 were at higher risk of CAD, whereas the CC genotype for rs4746720 of SIRT1 was associated with a significantly decreased risk of CAD. The A allele of the rs28365927 of SIRT3 showed a significant decreased risk association with CAD patient group (*P* = 0.014). Significant difference in genotypes rs350844 (G > A) (*P* = 0.004), rs350846 (G > C) (*P* = 0.002), and rs107251 (C > T) (*P* ≤ 0.01) for SIRT6 was also found between the CAD patients and the healthy controls. Haplotype CTA significantly increased the risk of CAD (*P* = 0.000118, OR = 1.497, 95%CI = 1.218–1.840), while haplotype GCG significantly decreases the risk of CAD (*P* = 0.000414, OR = 1.131, 95%CI = 0.791–1.619).

**Conclusions:**

The SNP rs28365927 in the SIRT3 gene and SNP rs350844, rs350846, and rs107251 in the SIRT6 gene present significant associations with CAD in a north Chinese population. Haplotype CTA and GCG generated by rs350846/rs107251/rs350844 in the SIRT6 might also increase and decrease the risk of CAD, respectively.

## 1. Introduction

The coronary artery supply blood, oxygen, and nutrients to the heart and coronary artery disease (CAD) occurs when the major blood vessels become damaged or diseased, manifested by stable angina, unstable angina, myocardial infarction (MI), or sudden cardiac death. Despite advanced medical and interventional therapies, CAD is still one of the main causes of morbidity and mortality, sparing no nation, ethnicity, or economic stratum [1, 2]. The main factors involved in the initiation and progression of CAD include inflammatory response, endothelial dysfunction, and metabolic dysregulation. Increasing researchers recently focus on genetic causes of CAD pathophysiological given that CAD is partly heritable. Genome-wide association studies (GWAS) have found the association for several gene variants with CAD [3, 4]. Large amount of efforts have been devoted to explore potential correlations between gene polymorphisms and the risk of CAD.

Sirtuins (silencing information regulators) are a highly conserved family of nicotinamide adenine dinucleotide- (NAD+-) dependent histone deacetylases, which contain seven members (SIRT1–SIRT7). The seven SIRTs are divided into four classes, as follows: class I (consisting of SIRT1, SIRT2, and SIRT3), class II (SIRT4), class III (SIRT5), and class IV (SIRT6 and SIRT7); they differ with respect to their distribution in tissues and their intracellular locations. SIRT1, SIRT6, and SIRT7 are predominantly classified in the nuclear. SIRT2 is localized predominantly in the cytoplasm, and SIRT3, SIRT4, and SIRT5 are located in the mitochondria. SIRTs play an important role in the development of cardiovascular diseases, including CAD, given their long known antiaging properties and the direct relation between aging and the deterioration of human organ systems [5, 6]. Studies have preliminarily revealed the relation between SIRTs and CAD. On the one hand, SIRTs are involved in a wide range of physiological functions, including calorie restriction [7], stress resistance [8], apoptosis, inflammation, mitochondrial function [9], and circadian clock [10], which are factors for CAD pathogenesis [11]. On the other hand, direct evidence has revealed the emerging role of SIRTs in mitochondrial dysfunction and cardiovascular diseases. Toulassi reviewed the protective role of SIRTs in atherosclerosis [12]. SIRT1 can inhibit oxidative stress and inflammation in patients with coronary artery disease [13]. SIRT3 also plays functional role on vascular biology and atherogenesis, and SIRT6 is a potential therapeutic target for cardiovascular diseases [14, 15]. As mentioned above, SIRTs mediated for CAD has been investigated in numerous aspects; however, the study about the role of SIRTs gene on CAD is limited.

Therefore, the present study assesses the association of several SIRT SNPs and the risk of CAD in a North Chinese population. Twelve SNPs (rs12778366 (T > C), rs3758391 (T > C), rs3740051 (A > G), rs4746720 (C > T), rs7895833 (G > A), rs932658 (A > C) for SIRT1, rs2015 (G > T) for SIRT2, rs28365927 (G > A), rs11246020 (C > T) for SIRT3, rs350844 (G > A), rs350846 (G > C), and rs107251 (C > T) for SIRT6) were selected for this study.

## 2. Materials and Methods

### 2.1. Study Subjects

A total of 509 patients with CAD and 552 healthy controls were recruited from the First Peoples' Hospital of Jining between April 2016 and September 2020. All enrolled CAD patients were evaluated by experienced cardiologists by coronary angiography due to significant coronary stenosis (≥50%) in at least one main coronary artery or their major branches (branch diameter ≥ 2 mm). Patients with previous history of coronary artery bypass graft surgery or a history of percutaneous coronary intervention were also included in CAD groups. Patients with serious heart failure, congenital heart disease, infectious heart disease, renal failure, diseases of the immune system, and malignant tumor were excluded. The 552 health controls matched with the patients in sex and age were enrolled at the same period, and they underwent the physical examination process by clinical examination and electrocardiogram. This study was designed according to the Declaration of Helsinki and was approved by the ethics committee of First Peoples' Hospital of Jining. All subjects provided written informed consents.

### 2.2. Genotyping and DNA Preparation

Approximately 1 ml venous blood was collected from the subjects, and SQ Blood DNA Kit II (D0714-250, Omega Bio-Tek, Norcross, UK) was used to extract and purify DNA according to the manufacturer's instructions. All DNA samples were genotyped using the polymerase chain reaction (PCR)–ligase detection reaction method. The PCR of the 12 target SNPs was amplified by the primers listed in Table 1 from each participant. A DNA sequencer was applied to detect the amplified products.

### 2.3. Statistical Analysis

All genotyping results from the studied patients and in controls were tested for Hardy–Weinberg equilibrium (HWE) by applying chi-square test (*χ*2 test). Chi-square statistics (*χ*2 test) were also used to compare the differences in genotype distributions and allele frequencies between CAD cases and control groups for statistical significance. The associations between the genotypes/alleles and CAD were evaluated via the odds ratio (OR), with a 95% confidence interval (CI) (95% CI). Pairwise linkage disequilibrium (LD) analyses were conducted using SHEsisPlus (http://shesisplus.biox.cn/SHEsis.html). A two-sided *P* value below 0.05 was considered statistically significant. All statistical analyses were performed with SPSS 17.0 for Windows (SPSS Inc., Chicago, IL, USA).

## 3. Results

No statistically significant differences were found between the CAD group and control group in terms of sex and age. The percentages of men and women were 47.2% and 52.8%, respectively, in the CAD groups and 46.9% and 53.1% in the controls (*P* = 0.953). The mean ± SD age was 56.99 ± 10.75 years for CAD group and 56.14 ± 11.40 years for control group (*P* = 0.212). The 12 observed genotype frequencies were in accordance with the Hardy–Weinberg equilibrium between the CAD group and control group (all *P* values were more than 0.05).

The details of genotype and allele of four gene polymorphism frequency distribution between CAD group and control group were compared, and the results are presented in Tables 2 and 3, respectively.

The wild homozygotes of TT genotype for rs3758391 and GG genotype for rs7895833 were at higher risk of CAD than the mutant heterozygote, with OR 2.352 (1.099–5.032) and 1.607 (1.015–2.544), respectively. The CC genotype for rs4746720 was associated with a significantly decreased risk of CAD (OR = 1.402, 95%CI = 1.018–1.932, *P* = 0.039) when compared with CT genotype. For rs28365927 of SIRT3, the A allele showed a significant decreased risk association with the CAD patient group (OR = 0.727, 95%CI = 0.564–0.937, *P* = 0.014).

Significant difference in genotypes rs350844 (G > A) (*P* = 0.004), rs350846 (G > C) (*P* = 0.002), and rs107251 (C > T) (*P* ≤ 0.01) for SIRT6 between the CAD patients and the healthy controls was also observed. The mutant homozygotes of AA genotype for rs350844 and CC genotype for rs107251 were at higher risk of CAD than the wild homozygote type, with OR 2.135 (1.315–3.469) and 2.114 (1.298–3.445), respectively. The A allele frequency for rs350844 and C allele frequency for rs350846 were significantly lower among the CAD cases than among the controls (*P* = 0.002 and *P* = 0.001, respectively). For rs107251, when the CC genotype was used as the reference, the CT, TT, and CT + TT genotypes were associated with a significantly increased risk of CAD (OR = 1.536, 95%CI = 1.194–1.976, *P* = 0.001 for CT vs. CC; OR = 2.010, 95%CI = 1.225–3.298, *P* = 0.006 for TT vs. CC; and OR = 1.594, 95%CI = 1.251–2.032, *P* ≤ 0.01 for CT + TT vs. CC). Moreover, the T allele showed a significant association with CAD patient group (OR = 1.439, 95%CI = 1.191–1.740, *P* ≤ 0.01).

**Figure 1:**
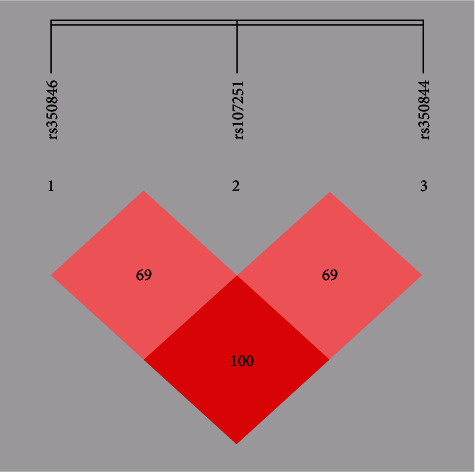
Linkage disequilibrium pattern between three SNPs, rs350846, rs107251, and rs350844, in CAD patients and healthy controls.

The linkage among the studied SNPs was investigated by haplotype analysis.  Figure 1 shows the LD block of rs350846/rs107251/rs350844 for SIRT6 gene; it presents a strong LD (rs350846/rs107251: D′ = 0.69, *r*^2^ = 0.46; rs107251/rs350844: D′ = 0.69, *r*^2^ = 0.46; rs350846/rs350844: D′ = 1, *r*^2^ = 0.97). The haplotype frequencies are presented in Table 4. The results demonstrate that haplotype CTA significantly increases the risk of CAD (*P* = 0.000118, OR = 1.497, 95%CI = 1.218–1.840), whereas haplotype GCG significantly decreases the risk of CAD (*P* = 0.000414, OR = 1.131, 95%CI = 0.791–1.619).

## 4. Discussion

In the present study, it investigated the association among 12 SNPs of SIRTs with CAD in a Chinese case–control study design with 509 CAD patient subjects and 552 healthy control subjects. The results demonstrated that rs28365927 for SIRT3 and rs350846/rs107251/rs350844 for SIRT6 showed a significant association with CAD.

SIRT1, which was the most widely studied member of sirtuin and participated in plenty of biological processes including cell cycle regulation, apoptosis, DNA repair and inflammation [16], autophagy, and aging [17], played an important role on the cardiovascular system. SIRT1 expression modulated a series of downstream cellular proteins, including its coactivator PGC-1*α*, PPAR-*γ*, the FoxO subgroup, AMPK, eNOS, protein tyrosine phosphatase, NF-*κ*B, and p53, which were vital regulators for cardiovascular system homeostasis. Studies have shown that SIRT1 deficiency contributed to increased inflammation, oxidative stress, foam cell formation, impaired nitric oxide (NO) production, and autophagy, thereby promoting vascular atherosclerosis [18], which is an important risk factor for CAD. One recent study by Ghaderian et al. [19] has shown that SIRT1 mRNA expression levels decreased in CAD patients with or without diabetes. This finding was consistent with an investigation that evaluated the protective effect of SIRT1 in predisposing CAD [13], showing that the expression of SIRT1 mRNA in patients with CAD decreased compared with controls. SIRT1 may play important effects on the development of CAD. The data from Li's group indicated that SIRT1 expression in peripheral blood mononuclear cells was significantly correlated with inflammatory cytokine levels in patients with CAD and type 2 diabetes but not with the severity of coronary lesions [20]. Rs7069102 was the most widely studied member of SIRT1 gene. Results from different research groups have demonstrated that rs7069102 was associated with the CAD risk; the G allele may increase CAD risk, but C allele may be protective against CAD [19, 21]. Potential mechanisms of rs7069102 were associated with the decreased expression of SIRT1, which may contribute to the CAD development through decreased ROS production. This study found that CT genotype for rs4746720 and GG genotype for rs7895833 were at higher risk of CAD. This finding was different from the results of other studies. For example, Cheng et al. [22] found no association of rs4746720 with MI risk, and Ramkaran et al. [23] reported no difference in the distribution of rs7895833 between South African Indian CAD patients and race-matched controls or black controls. This finding may have resulted from the population differences in our subjects that are mainly from North China. However, these studies recruited their volunteers from South China and South African.

Thus far, studies on the correlation between SIRT2 and CAD were relatively scarce. Reports indicate that SIRT2 could stabilize atherosclerotic plaques by preventing macrophage polarization to the M1 macrophage phenotype, which is crucial for atherosclerotic development [24]. Furthermore, SIRT2 showed prevention of obesity and metabolic diseases, which were important factors involved in CAD pathogenesis. Yang et al. [25] proposed that the SIRT2 gene promoter DNA sequence variants may alter transcriptional activity of SIRT2 and change SIRT2 level, contributing to myocardial infarction development as a rare risk factor. Studies demonstrated that the variant in rs2015 for SIRT2 gene was associated with susceptibility to colorectal cancer, Alzheimer's disease, and Parkinson's disease in Chinese Han population [26–28]. However, we did not find the correlation between rs2015 gene polymorphism and the susceptibility to CAD in this study.

SIRT3 was located in the mitochondrial matrix and highly expressed in tissues with high metabolic turnover and mitochondrial content, including the heart. The impact of SIRT in cardiac physiology and pathology has gained an increasing consideration in recent years and proved that impaired SIRT3 activity might play roles in the pathogenesis of various cardiac dysfunctions, such as cardiac hypertrophy, IR injury, and heart failure. Yin et al. [29] found that three SIRT3 gene variants, namely, rs11246029, rs71019893, and rs185277566, were more significantly frequent in MI (a specific type of CAD) patients than ethnic-matched healthy controls, indicating that SIRTs may contribute to the MI development as a risk factor. Consistently, our study presented that rs28365927 polymorphism of SIRT3 gene was associated with the risk of CAD. Coincidentally, volunteers collected by the two studies were from the same source of Chinese population in the same region in China. Thus, pursuing further exploration in a larger population from different regions is worthwhile.

SIRT6 affects the pathogenesis of various cardiovascular diseases by regulating cardiac hypertrophy, inflammatory, and oxidative stress. Studies showed that SIRT6 was highly relevant to the pathogenesis and progression of CAD and its complication disease. SIRT6 has been proposed to play protective roles in atherosclerosis, mainly depending on three approaches, namely, decreasing LDL cholesterol, reducing macrophage foam cell formation, or preventing endothelial dysfunction [30]. This study reported that the variant of the three selected SIRT6 genes, namely, rs350844, rs350846, and rs107251, was associated with the susceptibility to CAD in a Chinese population for the first time. By further linkage analysis, haplotype CTA and GCG of rs350846/rs107251/rs350844 for SIRT6 gene presented significant association with the risk of CAD. Some results of other studies were consistent with these results. For example, Tang et al. [31] found that two tag SNPs, rs352493 and rs3760908, within SIRT6 gene were associated with the severity of CAD in a Chinese Han population, and Wang showed that the two SNPs of SIRT6 gene promoter had significantly higher frequencies in MI patients than in controls, indicating that DSVs in MI patients may alter the transcriptional activity of the SIRT6 gene promoter and alter SIRT6 levels, thereby probably contributing to the risk of MI [32].

However, we were obliged to admit that there still existed certain shortcomings in this study. First, this study was only confined to a small population in northern China, and the sample size was limited. As we all know, genetic variations of different races may result in varying functions in different populations. Thus, larger sample size from different groups was required to substantiate the present findings. Second, the present study only included 12 genotypes of SIRT1, SIRT2, SIRT3, and SIRT6, which may not comprehensively evaluated the potential associations of SIRT polymorphisms with CAD. However, these limitations did not seem to affect the conclusion of this study. These findings promoted us to focus more on the association between SIRT6 gene polymorphisms and CAD in the future study.

In conclusion, we have investigated 12 SITRs SNPs with susceptibility to CAD in a north Chinese population through the present study. And we found significant associations of SNP rs28365927 in the SIRT3 gene and SNP rs350844, rs350846, and rs107251 in the SIRT6 gene with CAD in a north Chinese population. Haplotype CTA and GCG generated by rs350846/rs107251/rs350844 in the SIRT6 might also increase and decrease the risk of CAD, respectively. Although our data suggest a potential role of SIRTs polymorphisms in susceptibility to CAD, further studies involving a larger group of CAD patients and different ethnics were needed to conduct to consolidate these results.

## Figures and Tables

**Table 1:** Primers of SIRT genes used in the PCR.

	SNP	Ancestor allele	Primer sequence	Product size(bp)
SIRI1	rs12778366	T	F(5′-3′): ACGTTGGATGTAAGGCTTCTAGGACTGGAG R(5′-3′): ACGTTGGATGCTAAGGTCCTATCTACATCC	110
	rs3758391	T	F(5′-3′): ACGTTGGATGGCACACTGTGACTCCATATC R(5′-3′): ACGTTGGATGGCCATAACAAACACTGGCTC	100
	rs3740051	A	F(5′-3′): ACGTTGGATGAAAGGAGCCGCCTCCTTTTG R(5′-3′): ACGTTGGATGAGCTCCCTGAAATACGTTGG	97
	rs4746720	T	F(5′-3′): ACGTTGGATGGCCACAGTTTTGGAAAATGC R(5′-3′): ACGTTGGATGGTACTCAAAATCTGTTACGC	100
	rs7895833	G	F(5′-3′): ACGTTGGATGGTAATGAGGTGGTAAAAGGC R(5′-3′): ACGTTGGATGACTAGACAGGGCAGGATAAC	109
	rs932658	A	F(5′-3′): ACGTTGGATGGAATTTGGCTGCACTACACG R(5′-3′): ACGTTGGATGTGTTGCGTCTACCGCTCCG	117

SIRT2	rs2015	T	F(5′-3′): ACGTTGGATGACCTAACCTACCCCAGTGTG R(5′-3′): ACGTTGGATGTGAAGGCAGAGACTCGGGA	118

SIRT3	rs28365927	G	F(5′-3′): ACGTTGGATGTCCTTGCCCAAAATACCTCG R(5′-3′): ACGTTGGATGTTGCAGAGGCCTCCCAGAC	119
	rs11246020	C	F(5′-3′): ACGTTGGATGTTGTCATGAAGCAGCCGGAG R(5′-3′): ACGTTGGATGTTTCACTTTGGCCAAGGAGC	101

SIRT6	rs350844	A	F(5′-3′): ACGTTGGATGTCTGGGACATCGGATTCGAC R(5′-3′): ACGTTGGATGAAGTGTAAGACGTGAGTGCC	102
	rs350846	C	F(5′-3′): ACGTTGGATGACAACACAGCAAGTCAGAGG R(5′-3′): ACGTTGGATGTGGTGCGGTTCCGGGAAGAA	103
	rs107251	T	F(5′-3′): ACGTTGGATGTGACCAGAACTAGCACCCAG R(5′-3′): ACGTTGGATGGCTTTGTTTTCTGTGCTCCG	115

**Table 2:** Genotypic distribution of 12 SIRT gene between CAD patients (*n* = 509) and controls (*n* = 552).

	SNP	Genotype	Case (%)	Control (%)	*P* value^a^ (*χ*^2^)	*P* value^b^	OR (95% CI)
SIRI1	rs12778366	TT	382 (75.0)	426 (77.2)	0.222	Referent	1.00
		TC	116 (22.8)	121 (21.9)		0.651	1.069 (0.800-1.428)
		CC	11 (2.2)	5 (0.9)		0.099	2.453 (0.845-7.125)
		TC + CC	127 (25.0)	126 (22.8)		0.417	1.124 (0.847-1.491)
	rs3758391	TT	377 (74.1)	403(73.0)	0.030	Referent	1.00
		TC	110 (21.6)	139(25.2)		0.253	0.846 (0.635-1.127)
		CC	22 (4.3)	10(1.8)		0.028	2.352 (1.099-5.032)
		TC + CC	132 (25.9)	149 (27.0)		0.696	0.947 (0.721-1.244)
	rs3740051	AA	275 (54.0)	302(54.7)	0.139	Referent	1.00
		AG	192 (37.7)	221(40.0)		0.716	0.954 (0.741-1.229)
		GG	42 (8.3)	29 (5.3)		0.069	1.590 (0.964-2.624)
		AG + GG	234(46.0)	250 (45.3)		0.824	1.028 (0.807-1.309)
	rs4746720	CC	92 (18.1)	129 (23.4)	0.102	Referent	1.00
		CT	246 (48.3)	246 (44.6)		0.039	1.402 (1.018-1.932)
		TT	171 (33.6)	177 (32.1)		0.080	1.355 (0.964-1.903)
		CT + TT	417 (81.9)	423 (76.6)		0.034	1.382 (1.024-1.865)
	rs7895833	GG	270 (53.0)	292 (52.9)	0.051	Referent	1.00
		GA	187 (36.7)	225 (40.8)		0.412	0.899 (0.697-1.160)
		AA	52 (10.2)	35 (6.3)		0.043	1.607 (1.015-2.544)
		GA + AA	239 (47.0)	260 (47.1)		0.962	0.994 (0.781-1.265)
	rs932658	AA	364 (71.5)	383(69.4)	0.365	Referent	1.00
		AC	126 (24.8)	154(27.9)		0.287	0.861 (0.653-1.134)
		CC	19 (3.7)	15 (2.7)		0.416	1.333 (0.667-2.663)
		AC + CC	145 (28.5)	169 (30.6)		0.448	0.903 (0.693-1.176)

SIRT2	rs2015	GG	134 (26.3)	165 (29.9)	0.096	Referent	1.00
		GT	256 (50.3)	241 (43.7)		0.068	1.308 (0.981-1.744)
		TT	119 (23.4)	146 (26.4)		0.983	1.004 (0.720-1.399)
		GT + TT	375 (73.7)	387 (70.1)		0.197	1.193 (0.912-1.561)

SIRT3	rs28365927	GG	397 (78.0)	413 (74.8)	≤0.01	Referent	1.00
		GA	108 (21.2)	112 (20.3)		0.984	1.003 (0.745-1.351)
		AA	4 (0.8)	27 (4.9)		0.001	0.154 (0.053-0.444)
		CT + TT	112 (22.0)	139 (25.2)		0.224	0.838 (0.631-1.114)
	rs11246020	CC	397 (78.0)	417 (75.5)	0.009	Referent	1.00
		CT	105 (20.6)	135(24.5)		0.171	0.817 (0.611-1.091)
		TT	7 (1.4)	0 (0.0)		0.999	—
		CT + TT	112 (22.0)	135 (24.5)		0.345	0.871 (0.655-1.160)

SIRT6	rs350844	GG	224 (44.0)	287 (52.0)	0.004	Referent	1.00
		GA	235 (46.2)	235 (42.6)		0.053	1.281 (0.996-1.648)
		AA	50 (9.8)	30 (5.4)		0.002	2.135 (1.315-3.469)
		GA + AA	285 (56.0)	265 (48.0)		0.009	1.378 (1.082-1.755)
	rs350846	GG	224 (44.0)	296 (53.6)	0.002	Referent	1.00
		GC	237 (46.6)	226 (40.9)		0.011	1.386 (1.077-1.782)
		CC	48 (9.4)	30 (5.4)		0.003	2.114 (1.298-3.445)
		GC + CC	285 (56.0)	256(46.4)		0.002	1.471 (1.155-1.874)
	rs107251	CC	224 (44.0)	307 (55.6)	≤0.01	Referent	1.00
		CT	241 (47.3)	215 (38.9)		0.001	1.536 (1.194-1.976)
		TT	44 (8.6)	30 (5.4)		0.006	2.010 (1.225-3.298)
		CT + TT	285 (56.0)	245(44.4)		0.000	1.594 (1.251-2.032)

Abbreviations: CI: confidence interval; OR: odds ratio. ^a^*P* value for allele frequencies in cases and controls using 2-sided *χ*^2^ test. ^b^*P* values adjusted by age and gender using logistic regression. ^∗^*P* < 0.05.

**Table 3:** Allelic distribution of 12 SIRT gene between all CAD patients (2*n* = 1018) and controls (2*n* = 1104).

	SNP	Allele	Case (%)	Control (%)	*P* value^a^ (*χ*^2^)	*P* value^b^ (*χ*^2^)	OR (95% CI)
SIRI1	rs12778366	T	880 (86.4)	973 (88.1)	0.242	Referent	1.00
		C	138 (13.6)	131 (11.9)		0.243	1.165 (0.902-1.504)
	rs3758391	T	864 (84.9)	945 (85.6)	0.638	Referent	1.00
		C	154 (15.1)	159 (14.4)		0.638	1.059 (0.833-1.347)
	rs3740051	A	742 (72.9)	825 (74.7)	0.335	Referent	1.00
		G	276 (27.1)	279 (25.3)		0.335	1.100 (0.906-1.335)
	rs4746720	C	430 (42.2)	504 (45.7)	0.114	Referent	1.00
		T	588 (57.8)	600 (54.3)		0.114	1.149 (0.967-1.364)
	rs7895833	G	727 (71.4)	809 (73.3)	0.337	Referent	1.00
		A	291 (28.6)	295 (26.7)		0.337	1.098 (0.907-1.328)
	rs932658	A	854 (83.9)	920 (83.3)	0.729	Referent	1.00
		C	164 (16.1)	184 (16.7)		0.729	0.960 (0.763-1.209)

SIRT2	rs2015	G	524 (51.5)	570 (51.6)	0.996	Referent	1.00
		T	494 (48.5)	534 (48.4)		0.944	1.006 (0.848-1.193)

SIRT3	rs28365927	G	902 (88.6)	938 (85.0)	0.014	Referent	1.00
		A	116 (11.4)	166 (15.0)		0.014	0.727 (0.564-0.937)
	rs11246020	C	899 (88.3)	969 (87.8)	0.703	Referent	1.00
		T	119 (11.7)	135 (12.2)		0.703	0.950 (0.731-1.235)

SIRT6	rs350844	G	335 (32.9)	295 (26.7)	0.002	Referent	1.00
		A	683 (67.1)	809 (73.3)		0.002	1.345 (1.116-1.621)
	rs350846	G	333 (32.7)	286 (25.9)	0.001	Referent	1.00
		C	685 (67.3)	818 (74.1)		0.001	1.390 (1.152-1.678)
	rs107251	C	689 (67.7)	829 (75.1)	≤0.01	Referent	1.00
		T	329 (32.3)	275 (24.9)		0.000	1.439 (1.191-1.740)

Abbreviations: CI: confidence interval; OR: odds ratio. ^a^*P* value for allele frequencies in cases and controls using 2-sided *χ*^2^ test. ^b^*P* values adjusted by age and gender using logistic regression. ^∗^*P* < 0.05.

**Table 4:** Haplotype frequencies for *SIRT6* polymorphisms in CAD and control group.

Haplotype (rs350846/rs107251/rs350844)	CAD 2*n* = 1018 (%)	Controls 2*n* = 1104(%)	OR (95% CI)	*P* value
CCA	69 (6.8)	78 (7.1)	0.950 [0.679~1.329]	0.764414
CTA	264 (25.9)	208 (18.8)	1.497 [1.218~1.840]	0.000118^∗^
GCG	618 (60.7)	746 (67.6)	0.725 [0.606~0.867]	0.000414^∗^
GTG	65 (6.4)	62 (5.7)	1.131 [0.791~1.619]	0.499292

CAD: coronary artery disease; CI: confidence interval; OR: odds ratio. Haplotypes were omitted if the estimated haplotype frequency was <3%. ^∗^*P* < 0.05.

## Data Availability

Data generated during the study are available from the corresponding author by request.
